# Ionic liquid pretreatment of stinging nettle stems and giant miscanthus for bioethanol production

**DOI:** 10.1038/s41598-021-97993-y

**Published:** 2021-09-16

**Authors:** Małgorzata Smuga-Kogut, Daria Szymanowska-Powałowska, Roksana Markiewicz, Tomasz Piskier, Tomasz Kogut

**Affiliations:** 1 Department of Agrobiotechnology, Faculty of Mechanical Engineering, Koszalin University of Technology, Raclawicka 15-17, 75-620 Koszalin, Poland; 2 Department of Biotechnology and Food Microbiology, Poznan University of Life Sciences, Wojska Polskiego 48, 60-627 Poznan, Poland; 3 Department of Pharmacognosy, Poznan University of Medical Sciences, Swiecickiego 4, 61-781 Poznan, Poland; 4 NanoBioMedical Centre, Adam Mickiewicz University in Poznań, Wszechnicy Piastowskiej 3, 61614 Poznan, Poland; 5 Department of Geodesy and Offshore Survey, Maritime University of Szczecin, Żołnierska 46, 71-250 Szczecin, Poland

**Keywords:** Environmental biotechnology, Crop waste, Energy science and technology, Biofuels

## Abstract

Production of ethanol from lignocellulosic biomass is considered the most promising proposition for developing a sustainable and carbon–neutral energy system. The use of renewable raw materials and variability of lignocellulosic feedstock generating hexose and pentose sugars also brings advantages of the most abundant, sustainable and non-food competitive biomass. Great attention is now paid to agricultural wastes and overgrowing plants as an alternative to fast-growing energetic crops. The presented study explores the use of stinging nettle stems, which have not been treated as a source of bioethanol. Apart from being considered a weed, stinging nettle is used in pharmacy or cosmetics, yet its stems are always a non-edible waste. Therefore, the aim was to evaluate the effectiveness of pretreatment using imidazolium- and ammonium-based ionic liquids, enzymatic hydrolysis, fermentation of stinging nettle stems, and comparison of such a process with giant miscanthus. Raw and ionic liquid-pretreated feedstocks of stinging nettle and miscanthus were subjected to compositional analysis and scanning electron microscopy to determine the pretreatment effect. Next, the same conditions of enzymatic hydrolysis and fermentation were applied to both crops to explore the stinging nettle stems potential in the area of bioethanol production. The study showed that the pretreatment of both stinging nettle and miscanthus with imidazolium acetates allowed for increased availability of the critical lignocellulosic fraction. The use of 1-butyl-3-methylimidazolium acetate in the pretreatment of stinging nettle allowed to obtain very high ethanol concentrations of 7.3 g L^−1^, with 7.0 g L^−1^ achieved for miscanthus. Results similar for both plants were obtained for 1-ethyl-3-buthylimidazolium acetate. Moreover, in the case of ammonium ionic liquids, even though they have comparable potential to dissolve cellulose, it was impossible to depolymerize lignocellulose and extract lignin. Furthermore, they did not improve the efficiency of the hydrolysis process, which in turn led to low alcohol concentration. Overall, from the presented results, it can be assumed that the stinging nettle stems are a very promising bioenergy crop.

## Introduction

The increasing energy demand driven i.a. by economic growth, expanding population, and social pressure, is one of the most significant worldwide concerns, especially in the context of limited fossil fuel sources. The need to substitute fossil fuels is crucial to reach energy security and increase environmental sustainability^1^. Cellulosic ethanol is considered to also play an essential role in the creation of new technologies, since similarly to other industries (coal or corn processing), the development of cost-effective processes may induce diversification of products (other fuel molecules as well as various chemicals), leading therefore to a more sustainable chemical industry^2,3^. With the increase in technological possibilities, it is expected that the use of biomass for fuel purposes will soon increase to 388.6 (biomass of herbal origin) and 100.7 million tons of dry matter (wood biomass). When designing a process for sugar or ethanol production from biomass, its chemical composition must be taken into account, which varies according to the species of plant to be used in the production of bioethanol^4^. One of the crucial aspects of successful biofuel production is selecting a suitable source of biomass that will provide a large amount of cellulose and hemicellulose, a small amount of lignin, and will be easily purified during the chosen treatment. The use of biomass for bioethanol production is, in most cases, a very well-developed process. Nevertheless accessing biomass for chemical conversion requires complex evaluation of varieties of biomass like biomass size reduction, pretreatment, and fermentation. Moreover, the entire process should be reproducible, robust and able to convert closely related biomass source. Any new biomass source need to be evaluated carefully to determine preferred biochemical conversion schemes^5^.

From many advantages of bioethanol, one has to notice the possibility of its immediate use without the necessity to change its distribution and usage forms and carbon dioxide neutrality^6^. Naturally, some drawbacks of the production of bioethanol are also noted. As the first generation is based on edible crops, such as corn, sugar beet, or sugarcane, they threaten to maintain food security worldwide. The search for biomass for bioethanol production is ongoing to effectively replace fossil fuels and the future need for food demand. A good response for this problem is second-generation bioethanol, which is produced from non-edible crops feedstock materials, and include by-products (e.g., stems, leaves, and husks, wheat, rice or corn straws, sugar cane bagasse, forest residues), organic or municipal wastes, as well as dedicated, purpose-grown feedstocks (e.g., grasses, short-rotation forests, and other energy crops)^7–9^. Unfortunately, the increasing demand for non-food biomass may impact food security regarding food availability, diversity, and access^10^.

One of the most prevailing energy crops cultivated in a range of European and North American climatic conditions which can be used to produce bioethanol is giant miscanthus (*Miscanthus × giganteus*), since it has potential for greater photosynthetic efficiency and water and nitrogen use efficiency than other crops, especially when its production would take place on marginal lands with reduced input^11–13^. It has several advantages such as high cellulose content from 37 to 42% dry mass and high biomass yield per unit of planted area—23–38 Mg ha^−1^ year^−1^ under ideal conditions and 14–15 Mg ha^−1^ year^−1^ under poor conditions^12,14^. Lee and Kuan conducted a technical and economic analysis of bioethanol production from miscanthus, taking into account the costs of the following stages: pretreatment, enzymatic hydrolysis, and alcoholic fermentation and showed that the expected yield of ethanol from miscanthus is 250.0, 252.62, 255.80, 255.27 and 230.23 L per dry biomass in metric tons, and the corresponding ethanol costs are 0.891, 0.83, 0.88, 0.81 and 0.85 $ L^−1^ of ethanol in processes using AFEX pretreatment technologies, diluted acid, alkali, hot water, and steam explosion, respectively^15,16^. The results of these studies, similar to other research, show that the pretreatment process directly affects the price of the final product; therefore, it should yield as much fermented sugar as possible^15,17^.

A debate is still ongoing on energy crops, especially as they grow mostly on arable lands, reducing food-producing areas and increasing their prices. One way of resolving the food competitiveness problem is to promote feedstocks that can grow on marginal lands. The other is to use lignocellulosic biomass like agriculture residues, forest woody residues, microalgae, and even municipal solid wastes^18^. In this context, an interesting yet scarcely existing raw material for ethanol production literature is stinging nettle (*Urtica dioica* L.). It is a perennial, broadleaved, dioecious plant, reaching a height of 30 to over 100 cm found in temperate regions of Europe, Asia, North Africa, and North America^19^. Stinging nettle inhabits soils around houses, gardens, meadows, pastures, bushes, areas near lakes and rivers, and deciduous forests. It occurs in large groups on nitrogen-rich soils with very high phosphates content, with the yield reaching about 3–12 Mg ha^−1^ with relatively low inputs^20^. In intensive agriculture, stinging nettle is considered a weed. Bioethanol source might be considered significant because of its use as a potential, competitive with miscanthus in cellulose occurrence. Nettle can be used to produce high-quality agricultural raw materials for composites, medicine/pharmacy, textile, and energy sectors^20–22^. What is most important, stinging nettle stems always remains a waste, therefore the use of these parts of nettle for energy purposes doesn't involve the cultivation of stinging nettle intentionally for bioethanol production. Importantly, one need to notice the nettle stems may not be sufficient enough to replace the conventional energy crops, nevertheless in central Europe, they might serve as additional source for bioethanol production.

The overall efficiency of bioethanol production on a commercial basis will always consider sustainability, energy consumption, cost, and the overall efficacy of the methods applied^18^. A multistep biochemical process is used to produce bioethanol from lignocellulosic biomass, which usually involves raw material pretreatment, enzymatic hydrolysis, and ethanol fermentation^23,24^. Due to the complexity of the lignocellulosic complex (tight bonding and molecular packing of cellulose, hemicellulose and lignin, crystallinity of cellulose), it is necessary to pretreat the raw material to release the cellulose fraction, which will result in effective hydrolysis^25^. After pretreatment, complex compounds such as cellulose or hemicellulose are hydrolysed, and the released pool of fermenting sugars is metabolized to ethanol^17^.

Methods of lignocellulosic biomass pretreatment can be divided into various groups: physical, chemical, biological. In terms of chemical pretreatment, chemicals such as acids (sulfuric, hydrochloric, and phosphoric acids), alkali (NaOH, KOH, Ca(OH)_2_, hydrazine, and anhydrous ammonia) or organic solvents have been reported to have a meaningful effect on the structure of lignocellulose^26^. Such pretreatment methods may be characterized by drawbacks like high cost and energy demand, low yield of the process, or its unfavorability from the point of view of environmental impact. The combinatorial pretreatment (physicochemical and biochemical) and non-conventional technologies have been proposed, such as ultrasound, supercritical fluids, microwave irradiation, electric and/or magnetic fields^27^. Here, the most promising are Steam pretreatment, Liquid Hot Water pretreatment, Ammonia Fibre/Freeze Explosion, Organosolv or Ionic liquid (IL) based pretreatment, affecting physical and chemical properties of lignocellulose feedstocks^24–26^. Those pretreatment methods, similarly to the conventional ones, can have both advantages and disadvantages. Hydrothermal techniques, for example, are not appropriate for each lignocellulose biomass and usually are very energy-demanding. Nonetheless, they do not require using additional chemical reagents, which makes them environmentally friendly. On the other hand, ionic liquids emerged as lignocellulose pretreatment media thanks to solubilizing, fractioning and increasing cellulose enzymatic digestibility. During the IL pretreatment (dissolution and regeneration with anti-solvent), the crystalline structure of cellulose can be changed to amorphous, which largely increases the bioethanol production process efficiency^28–30^.

This work aimed to analyze the effectiveness of ethanol production from stinging nettle stems, an innovative cellulose source considered as agricultural waste. For this purpose, at first, pretreatment of stinging nettle stems was performed using imidazolium and ammonium ionic liquids. Afterward, the stems were subjected to enzymatic hydrolysis and alcoholic fermentation. As it is still challenging to compare the efficacy of bioethanol production from various lignocellulosic sources, the results obtained for stinging nettle stems were compared to the giant miscanthus, a well-known energy crop. The study's originality lies in the demonstration that the stinging nettle stems can be a potential raw material for ethanol production for fuel purposes.

## Results

### Compositional analysis

The qualitative composition of lignocellulose biomass is a crucial aspect that qualifies the raw material for bioethanol production. Another issue is the choice of appropriate pretreatment method and its costs. The use of ionic liquids has many advantages, such as the possibility of their recirculation and reuse and interesting physicochemical properties, e.g., low vapour pressure, thermal and chemical stability, and wide liquid range. The share of fractions of raw and IL-pretreated materials tested in the study is presented in Table 1.

**Table 1:** Composition of stinging nettle and giant miscanthus untreated and treated with ILs.

Sample	Pretreatment	Cellulose (%)	Hemicellulose (%)	Lignin (%)
*Urtica dioica* L.	Untreated	42.5 ± 0.5	18.7 ± 0.9	15.2 ± 0.9
	[bmim][OAc]	33.1 ± 0.7	22.3 ± 1.3	22.6 ± 0.7
	[emim][OAc]	35.8 ± 0.4	21.6 ± 2.7	15.1 ± 0.2
	[emim][DEP]	33.8 ± 1.9	21.1 ± 0.3	15.1 ± 0.2
	[CHDMA-C6][OAc]	43.4 ± 0.6	24.9 ± 0.8	22.3 ± 0.1
	[CHDMA-C4][OAc]	43.1 ± 0.2	24.8 ± 0.1	22.3 ± 0.2
*Miscanthus giganteus* (M × G)	Untreated	43.5 ± 1.1	25.7 ± 0.7	14.1 ± 0.1
	[bmim][OAc]	44.9 ± 0.5	31.6 ± 1.1	5.2 ± 0.1
	[emim][OAc]	47.7 ± 0.9	28.8 ± 1.0	5.5 ± 0.3
	[emim][DEP]	49.4 ± 0.3	21.9 ± 1.2	7.5 ± 0.2
	[CHDMA-C6][OAc]	46.0 ± 0.4	30.0 ± 0.3	16.4 ± 0.4
	[CHDMA-C4][OAc]	44.0 ± 0.3	31.6 ± 0.1	16.7 ± 0.1

Non-pretreated giant miscanthus contained on average 43.5% cellulose, 25.7% hemicellulose, and 14.1% lignin, while the stalks of stinging nettle contained 42.5% cellulose, 18.7% hemicellulose, and 15.2% lignin. The lignocellulose composition of these two plant species was similar in raw form. It differed depending on the type of ionic liquid used for its pretreatment. It should be pointed out that the pretreatment was carried out in two stages, which was also reflected in differences in the final composition of substrates aimed for hydrolysis. Stinging nettle stalks subjected to the influence of imidazolium ionic liquids caused a decrease in cellulose content by about 10% and lignin content by about 7–9%, with a simultaneous increase in the amount of hemicellulose. In turn, the pretreatment using ammonium ionic liquids did not affect carbohydrate losses and did not cause nettle stalk delignification. For comparison, similar results were obtained for giant miscanthus before and after pretreatment with ionic liquids. However, when imidazolium ionic liquids were used, the lignin content in miscanthus samples decreased to 5–7%, and the cellulose content increased by about 2–4%.

**Figure 1:**
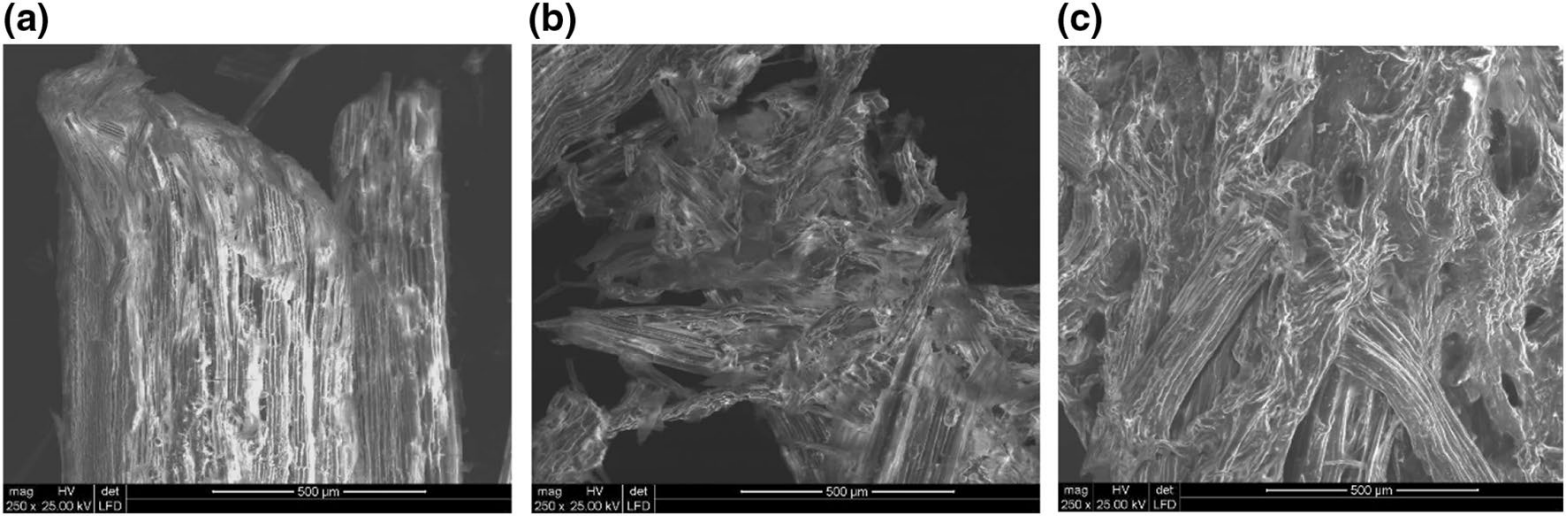
Scanning electron microscopic images of *Urtica dioica* L.: (**a**) untreated; (**b**) after pretreatment with [bmim][OAc]; (**c**) after pretreatment with [emim][OAc].

The use of imidazolium ionic liquids to dissolve miscanthus and nettle stalks, although it brings better results, is also troublesome. As cellulose dissolves, the liquid becomes more viscous and hardly miscible. When anti-solvent, in that case, deionized water is added, it turns difficult to dissolve the gel. Washing out cellulose fibres from the ionic liquid is challenging and time-consuming due to its dense consistency. This is not the case with ammonium ionic liquids. The viscosity of the ionic liquid and biomass solution was lower, which allowed further mixing. It was also easier to precipitate fibres from these liquids, and the process of ionic liquid washing out took a shorter time. Images from the scanning electron microscope exhibit that miscanthus and nettle cellulose fibres change from crystalline to amorphous forms after the treatment with both imidazolium and ammonium liquids, as presented in Fig. 1. On the other hand, the most significant disadvantage of ammonium ionic liquids is that using them is not possible to depolymerize lignocellulose and extract lignin.

### Enzymatic hydrolysis

**Figure 2:**
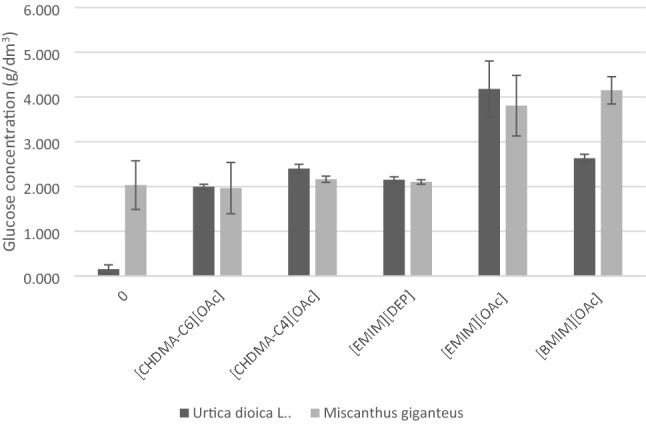
Glucose content after enzymatic hydrolysis of *Urtica dioica* L. and *Miscanthus × giganteus* untreated (0) and treated with an appropriate IL.

After pretreatment with ionic liquids, stinging nettle and giant miscanthus were subjected to enzymatic hydrolysis. The application of xylanase aimed to increase the porosity of cellulose fibres and increase the number of contact points for cellulolytic enzymes. The consequence of such action should be hemicellulose crystallization and exposure to cellulose fibres and, as a result, an increase in hydrolysis efficiency. The efficiency of enzymatic hydrolysis was evaluated after 96 h of the process, determining the glucose and xylose concentration. Glucose concentrations are presented in Fig. 2.

The highest concentration of glucose was determined in stinging nettle sample after the treatment with [emim][OAc] (4.5 g L^−1^) and in giant miscanthus after the treatment with [bmim][OAc] (4.1 g L^−1^) and [emim][OAc] (4.2 g L^−1^). In other cases, the glucose concentration did not exceed 2.5 g L^−1^, both for stinging nettle and miscanthus. Enzymatic hydrolysis occurred with very low efficiency in the variants where no treatment with ionic liquids was applied. The glucose concentration of 1.9 g L^−1^ was obtained for miscanthus and 0.025 g L^−1^ for stinging nettle.

**Figure 3:**
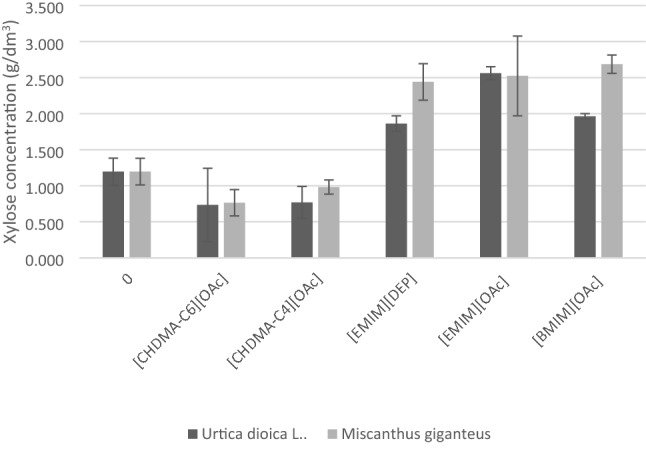
Xylose content after enzymatic hydrolysis of *Urtica dioica* L. and *Miscanthus × giganteus* samples untreated (0) and treated with an appropriate IL.

The content of xylose determined in samples subjected to enzymatic hydrolysis is presented in Fig. 3.

After application of [emim][OAc] pretreatment, 2.6 g L^−1^ and 2.5 g L^−1^ of xylose were obtained from stinging nettle and giant miscanthus, respectively. The xylose concentration in control samples did not exceed 1.5 g L^−1^. The lowest xylose content was observed in nettle and miscanthus samples treated with ammonium ionic liquids (results below 0.9 g L^−1^). Considering the influence of the application of individual ionic liquids on glucose and xylose content, it can be concluded that the most effective in the treatment of biomass is dissolution with [emim][OAc]. On the other hand, the ammonium ionic liquids did not improve the efficiency of the hydrolysis process in comparison to the native material.

### Alcoholic fermentation

**Figure 4:**
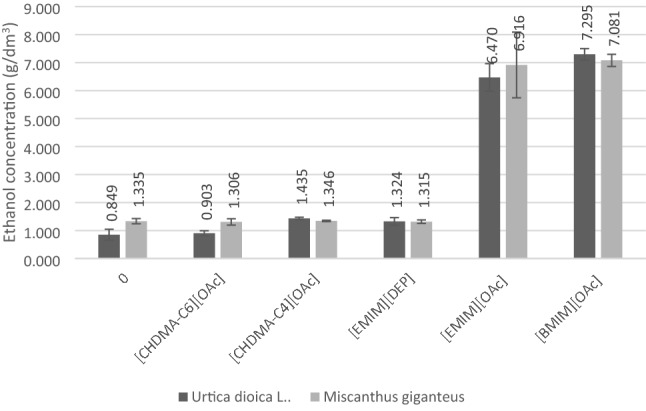
Ethanol content after alcoholic fermentation of *Urtica dioica* L. and *Miscanthus × giganteus* samples untreated and treated with ILs.

The obtained hydrolysates were subjected to alcoholic fermentation using *Saccharomyces cerevisiae* type II yeast. Chromatographic analysis showed that the highest concentration of ethanol was obtained in samples of stinging nettle (7.3 g L^−1^) and giant miscanthus (7.0 g L^−1^), which were pretreated with [bmim][OAc], as presented in Fig. 4.

**Figure 5:**
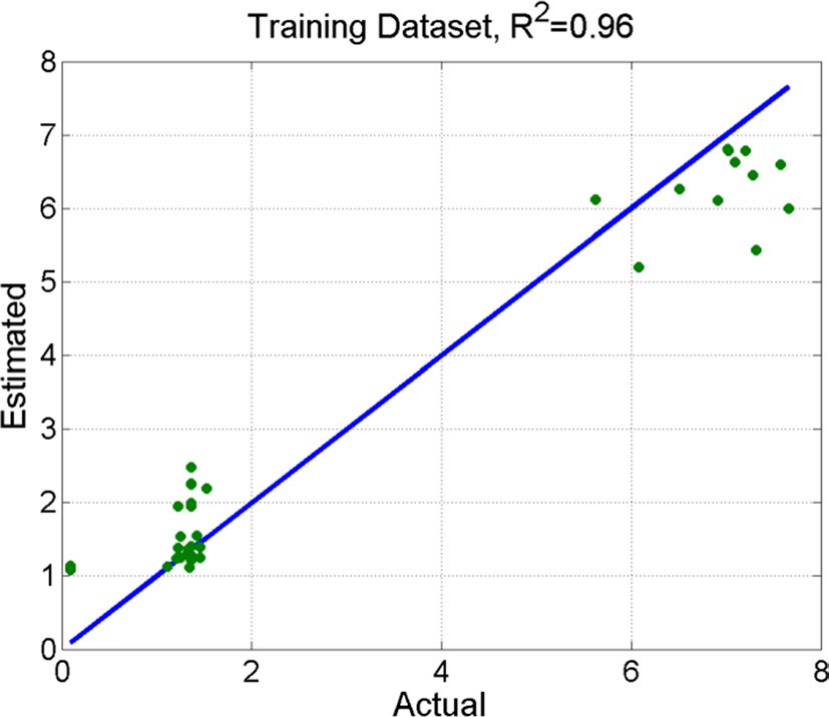
Verification scatter diagrams, with the x-axis showing the observed ethanol content and the y-axis presenting the estimated ethanol content.

The results of the statistical analysis include ordering the depending variables according to their level of significance. It was shown that the content of ethanol from biomass of stinging nettle and giant miscanthus is mainly affected by the type of ionic liquid used, then the amount of simple sugars after enzymatic hydrolysis, and the content of lignin in samples intended for hydrolysis and fermentation. Using the Random Forest machine learning algorithm, a model with a determination factor R2 between the estimated and the observed ethanol content of 0.96 was created, as presented in Fig. 5.

**Figure 6:**
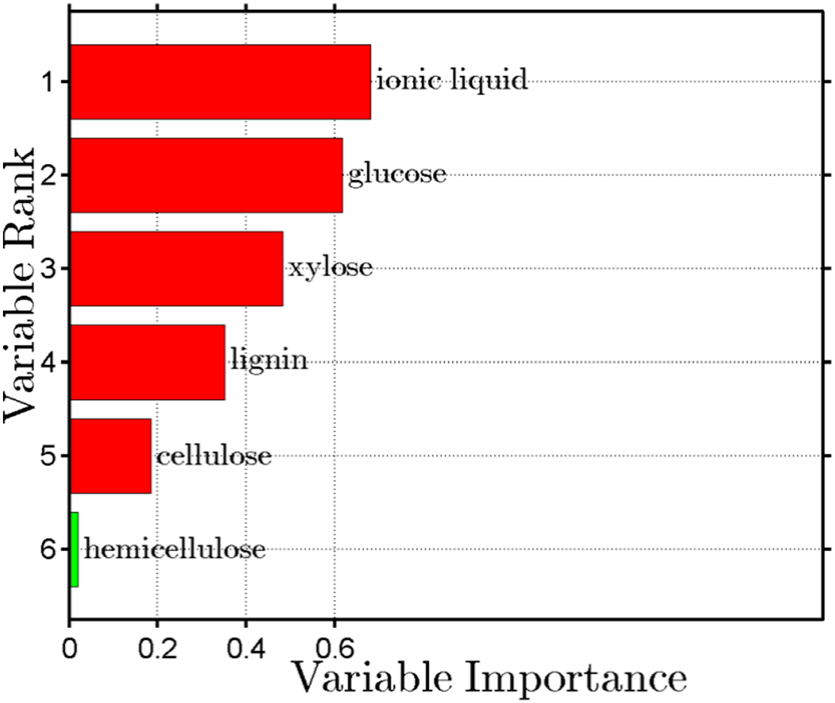
Relative importance of input features for estimating the ethanol content.

The collection of Out of Bag observations made it possible to determine the importance of particular traits for the content of ethanol from biomass. Information about the importance of a given variable is obtained directly from a trained model. Using the internal structure of the Random Forest algorithm, it is possible to determine how important are the traits used for its learning. In a stochastic manner, the algorithm selects the features of the model, thus estimating the significance of less important variables^31^. The traits ordered according to their significance in the model are shown in Fig. 6.

## Discussion

The production of ethanol from lignocellulose requires improvements and modifications related to pretreatment, enzymatic hydrolysis, and fermentation to increase the profitability of ethanol production and the transition from the laboratory to the industrial/commercial scale. One of the most important objectives is to increase the efficiency of the fermentation process so that the entire pool of sugars (pentose and hexose) is metabolized to ethanol.

Other barriers related to ethanol production include the variable composition of biomass, the presence of inhibitors as a result of pretreatment, osmotic and oxidative stress. However, the critical element in the production of second-generation bioethanol is the choice of raw material. The raw material from which bioethanol will be produced should contain the highest cellulose content possible, along with high hemicellulose and low lignin content, because it is this fraction of the lignocellulosic complex that is reflected in the concentration of fermenting sugars. A variety of lignocellulose feedstocks have been examined for use in the production of biofuels, including energy crops (e.g., miscanthus or switchgrass), forest-based woody wastes, and forest biomass, agricultural, industrial, municipal, and food wastes^32^. Another essential feature is the high availability of raw materials. Therefore, this article points to the possibility of using stinging nettle stems as a substrate for bioethanol production.

It was shown that the content of ethanol from stinging nettle and giant miscanthus biomass is affected mainly by the type of ionic liquid used for pretreatment, then the amount of simple sugars after enzymatic hydrolysis, and the content of lignin in samples intended for hydrolysis and fermentation. Importantly, not only the lignin content but also its structure influences bioethanol production. After the conventional pretreatment methods, more condensed lignin is generated, hampering the bioconversion efficiency^33^.

A pretreatment method well-suited to the raw material can significantly improve the hydrolysis efficiency of the lignocellulosic substrate. However, it should be pointed out that aggressive methods cause losses in the pool of fermenting sugars and contribute to the formation of process inhibitors^31^. Therefore, mild pretreatment methods are becoming more and more popular, which, as in the case of ionic liquids, can improve the availability of cellulose fibres and remove lignin but also causes the formation of large quantities of insoluble hemicelluloses^34–38^. Moreover, a combination of pretreatment methods is also gaining more and more attention^39^. The separation of lignin from cellulose using ILs depends on several factors. Hart et al. reported that hydrogen bonding strength was not a crucial factor for the lignin dissolution in ILs as it was in the cellulose dissolution, however a minimum hydrogen bonding basicity was still required to solubilize the lignin^40^. The removal of lignin in the pretreatment improves the efficiency of enzymatic hydrolysis but causes changes in the lignin structure. Moreover, even if lignin is not fully removed, its structural change also alters its position to cellulose fibres. It creates pores and free spaces, which ultimately causes the hydrolysis process to be more efficient. Unfortunately, there aren't many reports that would exhibit total fractionation of lignocellulose using ionic liquids into the main constituents. In this study, imidazolium ionic liquids ([emim][OAc], [bmim][OAc] and [emim][DEP]) along with ammonium ionic liquids ([CHDMA-C4][OAc] and [CHDMA-C6][OAc]) were chosen as the pretreatment agents for the raw materials due to their good cellulose solubility; [emim][OAc] allows the most effective dissolution of cellulose, from 8 to 10%, whereas [CHDMA-C6][OAc] and [CHDMA-C4][OAc] dissolve 9 and 7.5% of cellulose respectively^41–43^. An increase in cellulose content may be caused by the depolymerisation of fibres in ionic liquids, which improves their extraction. It can be assumed that the treatment with ammonium ionic liquids did not significantly influence the composition of the biomass studied (Table 1). In particular, it did not contribute to the removal of lignin, which makes it impossible to carry out further stages aimed at ethanol production effectively. Kumar et al. determined the content of individual lignocellulosic fractions after NaOH treatment (6–10%) for 24 h^44^. The authors showed a cellulose content of 85.93%, 6.8% hemicellulose, and 5.49% lignin. Agus Suryewan et al. used various pretreatment methods for the stinging nettle and obtained (in the best case studied) 85% of cellulose, 6% of hemicellulose, and 3% of lignin using water retting and decortication^22^.

Most of the ionic liquids used in biomass fractionation are imidazolium salts. The literature indicates that 1-ethyl-3-methylimidazolium acetate ([emim][OAc]), 1-allyl-3-methylimidazolium chloride ([amim][Cl]) and 1-butyl-3-methylimidazolium chloride ([bmim][Cl]) can serve as effective, non-derivatizing cellulose solvents at temperatures below 100 °C, and out of more than 20 ionic liquids tested, [amim][Cl] has proven to be an excellent wood chip solvent. For example, the addition of [bmim][Cl] causes the initial enzymatic hydrolysis rate and the pretreatment efficiency of the cellulose process to increase 50 times for regenerated cellulose compared to the untreated one^45^. Importantly, an increase in the rate of enzymatic hydrolysis of cellulose is associated with an increase in the production of simple sugars, which can be converted to ethanol. Moreover, the process of biomass degradation with the use of ionic liquids is less energy-intensive, easier to carry out, and more environmentally friendly than previously known solutions^24,28,46,47^. On the other hand, limitations for the application of ILs in the pretreatment of lignocellulose biomass are also being identified, with the most important factors being their high cost, high viscosities and moisture sensitivity, which makes it difficult to introduce an industrial scale process with their use^48^. For the process to be economically viable, water consumption must be reduced, and an effective system for ionic liquids recycling must be developed. Attempts were made to reduce the cost of solvent acquisition, replacing imidazolium ionic liquids with liquid obtained from aromatic aldehydes of lignin and hemicellulose, i.e., by-products from biofuel production^49,50^. The results were similar, although the reaction with [emim][OAc] was slightly slower. The best results obtained for the ionic liquid [emim][OAc] are explained in the literature. They are related to acetate ([OAc]) anion, which was demonstrated to be efficient in the dissolution of lignocellulosic biomass^51^.

It was reported that both imidazolium and ammonium ionic liquids compete for hydrogen bonds present in cellulose structure, thus disrupting its three dimensions network^26,41^. It was reported that a key reason for this was the high hydrogen bond acceptor capacity (β) of the [OAc] anion (β = 1.201) in comparison to previously mentioned chloride anion (β = 0.83)^52^. Due to this, 1-ethyl-3-methylimidazolium acetate is confirmed to be one of the best and is one of the most commonly used ILs, able to dissolve a large variety of lignocellulosic biomass and to fractionate it into cellulose-and hemicellulose-rich fractions, as well as to produce high pure lignin^53–55^. In the case of the presented cyclohexylammonium ionic liquids, the high performance of [CHDMA-C6][OAc] affected all alkyl groups' fine-tunning. According to the mechanism described previously, two activity categories were fundamental: (1) two methyl groups using its six activated C–H bonds to link with both the acetate and cellulose surface and (2) hexyl and especially cyclohexyl are symmetry breaking substituents. Similarly, as in the case of imidazolium ionic liquids, appropriate cation allows exploiting proton acceptability of carboxylate, which further enables the breakdown of inter-and intramolecular hydrogen bonds^41^.

The loosening of the lignocellulosic complex structure significantly facilitates enzymatic hydrolysis, the effectiveness of which depends on the selection of enzymatic preparations. Studies on the hydrolysis of a specific raw material are closely related to optimizing the preparation dose and process conditions. These arduous activities are usually carried out on selected 2–3 variants, characterized by the highest cellulose concentration after pretreatment. At the initial stage of research on the suitability of a given raw material for ethanol production, it is advisable to select enzymatic preparations known and tested in the context of hydrolysis effectiveness. However, it is worth mentioning that an important element of the lignocellulosic complex is hemicellulose, which may significantly reduce the effectiveness of hydrolysis^56^. Therefore, it is justified to use xylanases, which increase the material's porosity, expose cellulose fibres, and result in higher concentrations of fermenting sugars, which was also performed in this study (Figs. 2 and 3) is justified in the literature^57,58^. The next stage of second-generation bioethanol production is ethanol fermentation. In this study, the biosynthesis of ethanol was carried out with the participation of *Saccharomyces cerevisiae* yeast. Hydrolysates from giant miscanthus and stinging nettle were compared.

The full potential of stinging nettle has not yet been shown in any experimental work, and importantly it was not without reason that we have decided to use a plant that has not yet been used for ethanol production and compare it with one of the most popular energy plants used in this context. The production of ethanol from giant miscanthus has already been the subject of many studies comparing the methods and effectiveness of pretreatment, the degree of hemicellulose conversion to fermenting sugars, and the efficiency of ethanol fermentation.

This study focused on the potential of novel lignocellulosic wastes and their comparison at the same conditions applied to well-known biomass sources. The ethanol concentration obtained for both investigated raw materials is comparable, as presented in Fig. 4, which means that the stinging nettle stems are a promising alternative to energy crops such as giant miscanthus.

## Materials and methods

### Raw material

Common nettle stalks used for the research came from an agricultural wasteland with an area of 4.9 ha (Maszkowo, Zachodniopomorskie, Poland) excluded from agricultural production for 15 years. The plant was identified by Tomasz Piskier based on a plant atlas. The giant miscanthus was obtained from the resources of the Department of Agrobiotechnology (Koszalin University of Technology). Both plants were obtained under the principles of due carefulness included in the provisions of the Regulation (EU) No. 511/2014 of the European Parliament and of the Council (April 16, 2014). As both plants were collected from the territory of Poland, they are not subjected to the provisions on genetic resources of the previously mentioned Regulation No. 511/2014 and suitable permission of their use has been obtained.

Dry stalks of stinging nettle were cut down after the vegetation period (in September 2017), at the height of about 10–15 cm above the ground, then dried to a moisture content below 10% and ground in a colloidal mill up to 1 mm in size. A similar procedure was applied to the aboveground parts of giant miscanthus harvested in September 2017.

### Ionic liquids

**Figure 7:**
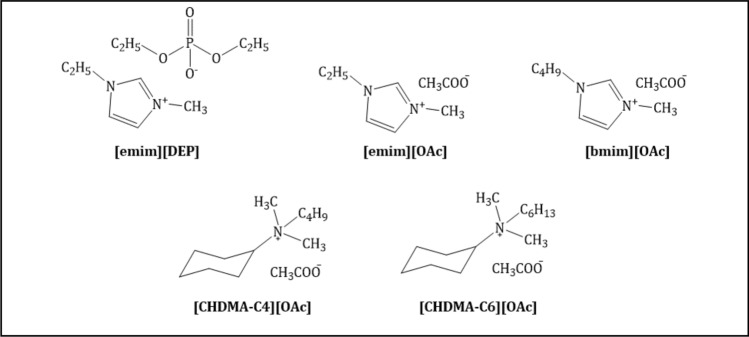
Ionic liquids chosen for pretreatment of stinging nettle stems and giant miscanthus.

For the pretreatment of cellulose-rich material, five ILs from imidazolium and ammonium groups were chosen, as presented in Fig. 7. Three of them were commercially available (Iolitech GmbH, Germany) imidazolium ILs: 1-ethyl-3-methylimidazolium acetate ([emim][OAc]), 1-butyl-3-methylimidazolium acetate ([bmim][OAc]) and 1-ethyl-3-methylimidazolium diethyl phosphate ([emim][DEP]). The remaining two, belonging to the group of ammonium ILs, namely butyl(cyclohexyl)dimethylammonium acetate (([CHDMA-C4][OAc]) and (cyclohexyl)hexyldimethylammonium acetate ([CHDMA-C6][OAc]), were synthesized according to already established protocols^41,59^.

### Raw material pretreatment

10 g of ground stalks of stinging nettle and giant miscanthus were added to 50 cm^3^ of an appropriate ionic liquid, homogenized, and dissolved at 120 °C for 2 h. After incubation, the samples were left to cool, and then deionized water was added to rinse the cellulose fibres and separate the biomass from the IL. During the addition of the deionized water, the IL dissolves in water, and the plant fraction precipitates. The water-IL solution with biomass was filtered on a Shot funnel with a filter (Whatman 1.0 paper). This procedure was repeated four times for imidazolium ILs, where a significant increase in the plant-IL mixture was present, and two times for ammonium ILs. Such purified stalks of nettle and miscanthus were subjected to enzymatic hydrolysis.

### Enzymatic hydrolysis

Thermostable xylanase, derived from a modified strain of *E. coli* bacteria (Sigma Aldrich) and CellicCTec2 enzyme were used for enzymatic hydrolysis of biomass samples (purified and non-purified ones). The initial cellulose and hemicellulose concentration was 1.0% (w/v) based on 100 mL (50 mM sodium citrate buffer) of total liquid in 250 mL Erlenmeyer flasks. Initially, xylanase (≥ 40 units mg^−1^) in the amount of 8 U mg^−1^ hemicellulose was added and incubated at 65 °C, pH 5.0. Hydrolysis at this stage was carried out for 24 h with 250 rpm mixing. After this time, the temperature was lowered to 50 °C, and 15 FPU g^−1^ cellulose of commercial cellulase enzyme Cellic CTec2 (Novozymes, Denmark) was added to the solutions. After 96 h of enzymatic hydrolysis with the use of cellulases complex, the content of glucose and xylose was determined with the use of high-performance liquid chromatography. All experiments were performed three times to establish a standard deviation.

### Alcoholic fermentation

The alcoholic fermentation was carried out accordingly to our previous reports^59–61^. Hydrolysate solutions, previously filtered to separate the lignocellulose residue, were subjected to alcoholic fermentation. The pH of the fermentation broth was measured at each sampling and adjusted to 5.0 by the addition of either 10 wt% H_2_SO_4_ or 20 wt% NaOH. Fermentation was started by adding freeze-dried distiller's yeast *Saccharomyces cerevisiae* type II (Sigma-Aldrich) (5% v/v). Ethanol fermentation was conducted for four days in anaerobic conditions. Samples were taken and analysed for ethanol concentration after fermentation.

### Analytical methods

To examine the influence of ionic liquids on the structure of lignocellulose and the amount of available cellulose, all samples were tested for the content of cellulose, lignin, and hemicellulose (Ankom A200; ANKOM Technology); the crystalline structure of the samples was recorded using a scanning electron microscope (SEM). The morphology of cellulose fibers in buckwheat straw samples before and after ionic liquid pretreatment was recorded using SEM FEI Quanta 200 Mark 2. The content of glucose and ethanol was determined by using high-performance liquid chromatography. Samples were first centrifuged at 4000×*g* for 10 min at 4 °C (Multifuge 3SR, Germany) and then was filtered through a 0.22 µm membrane filter (Millex-GS, Millipore, USA) before analysis using an HPLC system (Merck Hitachi, Germany). Glucose and ethanol were separated on an Aminex HPX-87P (Bio-Rad, USA) at 30 °C using a 5 mM H_2_SO_4_ as the mobile phase at a flow rate of 0.6 cm^3^ min^−1^ and then detected with a refractive index detector (Model L-7490, Merck Hitachi, Germany). All the analytical methods have been described in detail before in our previous works^59–61^.

The Random Forest algorithm implemented by David Lary (https://davidlary.info) in Matlab was used to analyze the results. The Random Forest algorithm generalizes the idea of decision trees and is based mainly on the bagging method. The concept of this algorithm is based on the construction of a group of decision trees, which are created based on a random data set^62^. Classification in this algorithm is based on the voting of classifiers. The assessment of the probability of misclassification, built into the mechanism of the classifier, allows determining the out of bag error (OOB). Thanks to OOB observations, it is also possible to estimate the importance of the observation vector variables from the point of view of the classification^63^ based on this property, the vector of traits (f) was constructed, based on which their significance in the process of bioethanol production was determined, as presented in the Eq. (1),

1 $${\mathbf{f}} \, = \, \left[ {{\varvec{G}}, \, {\varvec{X}}, \, {\varvec{C}}, \, {\varvec{L}}, \, {\varvec{H}}, \, {\varvec{IL}}} \right]^{{\mathbf{T}}} ,$$

where G is the glucose; X is the xylose; C is the cellulose; L is the lignin; H is the hemicellulose; IL is the ionic liquid.

## Conclusions

Pretreatment of stinging nettle and giant miscanthus with imidazolium ionic liquids allow for the increase of the availability of a key fraction of lignocellulose which is cellulose. Such application of [bmim][OAc] in the pretreatment of stinging nettle stems (and subsequent enzymatic hydrolysis) allowed us to obtain the highest concentrations of ethanol in the fermentation process, equal to 7.3 g L^−1^. In comparison, the ethanol amount achieved for miscanthus was 7.0 g L^−1^. Moreover, it was shown that ammonium liquids, although they allow for the more effective dissolution of the raw material, do not increase the concentration of ethanol in the fermentation process. Given the presented results of bioprocesses conducted and literature data related to the common occurrence and characteristics of the raw material, it can be assumed that stinging nettle, which in the case of the used stems is considered an agricultural waste, is a promising raw material for the production of second-generation bioethanol.
